# Serum YKL-40 Levels, Leukocyte Profiles, and Acute Exacerbations of Advanced COPD

**DOI:** 10.3390/jcm12186106

**Published:** 2023-09-21

**Authors:** Romana Olivia Popețiu, Imola Donath-Miklos, Simona Maria Borta, Larisa Alexandra Rus, Anamaria Vîlcea, Dragoș Vasile Nica, Maria Pușchiță

**Affiliations:** 1 Department of Internal Medicine, Faculty of Medicine, “Vasile Goldiș” Western University of Arad, Bulevardul Revoluției 94, 310025 Arad, Romania; simoborta@yahoo.com (S.M.B.); larisa_gal@yahoo.com (L.A.R.); anamariavilcea33@gmail.com (A.V.); mpuschita.mp@gmail.com (M.P.); 2 Arad County Emergency Clinical Hospital, Str. Andrényi Károly Nr. 2-4, 310037 Arad, Romania; 3 Department of Physiology, Faculty of Medicine, “Vasile Goldiș” Western University of Arad, Bulevardul Revoluției 94, 310025 Arad, Romania; miklosimola@gmail.com; 4 The National Institute of Research-Development for Machines and Installations Designed for Agriculture and Food Industry, Bulevardul Ion Ionescu de la Brad 6, 077190 București, Romania; nicadragos@gmail.com; 5 Research Center for Pharmaco-Toxicological Evaluations, Faculty of Pharmacy, “Victor Babes” University of Medicine and Pharmacy, Eftimie Murgu Square No. 2, 300041 Timişoara, Romania

**Keywords:** advanced COPD, YKL-40, complete blood count, leukocyte profiles, inflammatory environment

## Abstract

Little information exists on YKL-40—a key protein in tissue remodeling—and complete blood count (CBC) parameters during acute exacerbations of advanced chronic obstructive pulmonary disease (COPD). This pilot exploratory study (August 2020–January 2021) investigated the connection between serum YKL-40 levels and CBC profile in sex- and age-matched individuals with severe COPD (GOLD stage III, *n* = 23, median age = 66 years, 65.21% males) and very severe COPD (GOLD stage IV, *n* = 24, median age = 66.5 years, 74.81% males). The measured parameters were serum YKL-40, absolute leukocyte count (ALLC), absolute neutrophil count (ANC), neutrophil percentage, absolute lymphocyte count (ALC), lymphocyte percentage, neutrophil-to-lymphocyte ratio (NLR), absolute eosinophil count (AEC), eosinophil percentage, absolute monocyte count (AMC), monocyte percentage, absolute basophil count (ABC), basophil percentage, hemoglobin levels, and hematocrit concentrations. No significant inter-group differences were observed. However, high YKL-40 subjects (*n* = 23)—as stratified via median YKL-40 (3934.5 pg/mL)—showed significantly increased neutrophil percentage and NLR but significantly lower lymphocyte-, eosinophil-, and basophil-related parameters compared to low YKL-40 patients (*n* = 24). These results reveal multidimensional, YKL-40-associated changes in leukocyte profile of patients with advanced COPD during acute exacerbations, with potential implications for personalized treatment.

## 1. Introduction

With a rising incidence and prevalence during the past few decades (≈300 million people and ≈12.2%, respectively, in 2020), chronic obstructive pulmonary disease (COPD) is a significant global health problem [1]. This respiratory disorder is associated with an important socioeconomic burden on patients, their caregivers, and healthcare systems [2]. Acute exacerbations of COPD (AECOPDs) are important factors contributing to this burden [3]. Mainly caused by bacterial/viral infections and environmental stresses, these episodes of worsening respiratory symptoms are characterized by increased breathlessness, cough, and wheezing, leading to frequent hospital (re)admissions and elevated healthcare costs [4]. The incidence of AECOPDs increases with COPD severity [4], and consequently, patients with advanced disease—i.e., GOLD stage III (herein named severe COPD) and GOLD stage IV (herein named very severe COPD)—represent a cluster of patients with special needs [5].

Primarily associated with smoking and age above 40 years [6], COPD is characterized by low-grade, chronic inflammation of the airways and lung parenchyma resulting in progressive, poorly reversible airflow limitation [7,8]. Another key aspect of COPD pathophysiology is airway remodeling related to epithelial cell dysfunction, submucosal gland hypertrophy, smooth muscle hypertrophy/hyperplasia, airway fibrosis, mucus hypersecretion, increased angiogenesis, and dysbalance of matrix metalloproteinases and their tissue inhibitors [6]. However, the standard method used in clinical practice for COPD diagnosis, staging, and monitoring—i.e., spirometry [9]—does not provide information on airway inflammation or pathophysiological processes underlying its development and clinical course [10]. Numerous studies hence aimed at the identification of novel biomarkers for this respiratory disorder [6,10].

During the last decade, YKL-40 has gained increased recognition as a potential biomarker for COPD-related airway inflammation [7,8,11,12,13,14,15]. This 40 kDa glycoprotein is produced by various cell types, including neutrophils, macrophages, chondrocytes, vascular smooth muscle cells, and cancer cells [7,13]. Despite not having a fully clarified function, accumulating evidence suggests that YKL-40 is central to key cellular processes (e.g., growth, chemotaxis, and migration) and tissular events (e.g., remodeling)—as reviewed by Tong et al. (2018) [7]. This polypeptide also correlates directly with COPD severity and AECOPD frequency [7,8,14,15,16]. However, up-to-date studies have addressed the relevance of YKL-40 in AECOPDs using cohorts of patients covering all stages of COPD [7,8] but did not focus on individuals with advanced disease despite their increased morbidity and mortality risk [5].

As a routine, inexpensive, and easily available medical laboratory test, the complete blood count (CBC) is a valuable aid in determining the risk, diagnosis, and prognostic of various health conditions, including respiratory diseases [17,18]. The absolute number and frequency of different types of white blood cells (e.g., neutrophils, lymphocytes, monocytes, eosinophils, and basophils) or derived parameters (such as the neutrophil-to-lymphocyte ratio (NLR) or the monocyte-to-basophil ratio (MLR)) can serve as pertinent—although etiologically non-specific—inflammation biomarkers [18]. A growing body of clinical data supports the potential use of such parameters as biomarkers for AECOPD severity and (re)admission risk [8,15,18,19,20,21]. However, there is no targeted investigation on CBC relevance during exacerbations of advanced COPD. There is also little information connecting YKL-40 and CBC parameters during AECOPDs [15,18]. Moreover, no study has investigated these relationships in adults with advanced COPD.

The present study was conducted in a cohort of age- and sex-matched patients with advanced COPD. We hypothesized that individuals with different serum YKL-40 levels may exhibit different CBC profiles during AECOPDs. Firstly, we compared the expression of serum YKL-40 and CBC parameters in these subjects stratified based on the COPD stage (severe COPD vs. very severe COPD). Next, we investigated the differences in CBC-related markers for different YKL-40 strata. The analyzed variables included 14 CBC-related parameters, more precisely the absolute leukocyte count (ALLC), the absolute neutrophil count (ANC), the neutrophil percentage, the absolute lymphocyte count (ALC), the lymphocyte percentage, the neutrophil-to-lymphocyte ratio (NLR), the absolute eosinophil count (AEC), the eosinophil percentage, the absolute monocyte count (AMC), the monocyte percentage, the absolute basophil count (ABC), the basophil percentage, hemoglobin levels, and hematocrit concentrations.

## 2. Materials and Methods

### 2.1. Study Design

An exploratory pilot single-site investigation using a retrospective cross-sectional design was performed at the Department of Pneumology of the Arad County Emergency Clinical Hospital (Arad, Romania) between August 2020 and January 2021. Located in the largest city in western Romania (the city of Arad), this second-level hospital is a multipurpose facility with 1322 beds, making it one of the largest hospitals in Romania [22]. It provides care for over 400,000 people, serving all of Arad County and its neighboring counties [22]. The study was approved by the ethics committees (IECs) of the two institutions involved, namely the Arad County Clinical Hospital (approval No. 26/29 October 2019) and the “Vasile Goldiș” Western University of Arad, Romania (approval No. 159/12 December 2019). Ethical approval was in accordance with the Declaration of Helsinki of 1964 and later amendments. All patients or their caregivers received and signed an informed consent. All information related to the patients’ identification data was strictly protected.

### 2.2. Patients and Measurements

All subjects were hospitalized due to AECOPDs. An exacerbation was defined as “an acute worsening of respiratory symptoms that results in additional therapy” as per the 2023 GOLD report [23]. The initial pool of patients included both cases previously known to have severe COPD or very severe COPD (herein grouped together under the term advanced COPD) and cases newly diagnosed with advanced COPD. This category of patients displays a more compromised lung function and experiences more frequent AECOPDs, has an elevated mortality/morbidity, and requires higher healthcare utilization compared to milder forms of the disease (i.e., GOLD stage I and II) [1,5,6,12,23]. If an intervention is useful for reducing these resource-intensive events, it could lead to cost savings for healthcare systems and payers. Selecting only advanced COPD patients for clinical studies therefore has the potential to provide more focused and relevant data for the development of treatments targeting this high-risk population. Moreover, by focusing on advanced COPD patients, the sample size required for a trial may be smaller, reducing the overall costs and allowing the trial to be conducted more quickly.

For the former category of patients, the diagnosis and staging of COPD were previously conducted by a pulmonary specialist in our center via spirometry according to the criteria of the Global Initiative for Chronic Obstructive Lung Disease guidelines—FEV1/FVC ratio <70% for COPD diagnosis and FEV1 = 30–49% for diagnosing severe COPD (*syn.* GOLD stage III) or FEV1 < 30% for diagnosing very severe COPD (*syn.* GOLD stage IV) [23]—and taking into account onset after age 40; prolonged exposure to risk factors (e.g., tobacco smoke, biomass fuels); history or physical evidence of dyspnea, cough, sputum, and wheezing; persistent, progressively worsening symptoms despite treatment; impaired lung function between symptoms; limited relief from rapid-acting bronchodilator treatment; and severe hyperinflation or other changes on chest X-ray. In the case of new patients, the diagnosis and staging were conducted in a similar manner at the moment of their hospitalization. Patients with concurrent disease(s), e.g., pneumonia, asthma, rhinitis, lung cancer, interstitial lung diseases, and rheumatologic, liver, renal, and neurological muscular diseases that may have caused acute respiratory symptoms; receiving systemic corticosteroids in the last four weeks, antibiotics for lower respiratory infection during the past six weeks, or β-blockers; or with known hypersensitivity to salbutamol or a history of tachyarrhythmia were excluded from the study [6].

To provide us with a homogeneous study group, the patients were selected after age- and sex-matching from an initial cohort of 98 individuals. The total pack-year smoking history was calculated for each subject based on their responses at the enrollment time. Because the present trial was run during the COVID-19 pandemic (from August 2020 to January 2021), accessing a larger pool of adults with advanced COPD was not possible. The absence of an electronic database of spirometry values did not allow us to detail data about the pulmonary function of the enrolled patients. However, we checked all subjects’ findings if they appeared suspicious.

The recommended therapies were homogeneous within the two groups. Blood sampling was performed during the exacerbation as soon as possible after hospital admission. These samples were used to determine the serum YKL-40 levels, ALLC, ANC, neutrophil percentage, ALC, lymphocyte percentage, AEC, eosinophil percentage, AMC, monocyte percentage, ABC, basophil percentage, hemoglobin levels, and hematocrit concentrations (triplicate analysis per each sample). CBC analyses were conducted at the Arad County Emergency Clinical Hospital using a Sysmex XN-1500 Hematology Analyzer (Sysmex Corporation, Kobe, Japan).

YKL-40 measurements were conducted at the “Vasile Goldiș” Western University of Arad using the Human Chitinase-3-like Protein 1 ELISA Kit (code CSB-E13608h; Cusabio Technology LLC, Houston, TX, USA). This kit is fast (1–5 h) and has a good sensitivity to human YKL-40 in serum, plasma, and tissue homogenates; that is, 78.065 pg/mL, with a detection range between 46.875 and 3000 pg/mL—which covers the range of normality in humans [7,8]. In addition, the antibodies used (CHI3L1 antibodies) have been validated for human use and ELISA [24]. The cost per sample (in our case) was about EUR 30–35. Although this parameter is not routinely determined in clinical practice, we thought that adding this variable to the traditional approach in COPD diagnosis, monitoring, and treatment (e.g., clinical evaluation, spirometry, imaging studies, and symptom assessment) may have utility in improving treatment decisions, since this protein is closely connected to inflammation and tissue remodeling processes in COPD [7,8,11,12,13,14,15]. Thus, high YKL-40 individuals might benefit from more aggressive interventions to mitigate the impact of AECOPDs. Identifying subpopulations of advanced COPD patients with specific biomarker profiles, including YKL-40, may also help researchers design targeted therapies and conduct clinical trials more efficiently. Moreover, it may help healthcare systems improve resource allocation. For example, a decrease in post-treatment serum YKL-40 levels may indicate a positive response, while persistently high levels might suggest the need for further intervention or closer monitoring.

Both samples and standards were run in triplicates. Briefly, 100 microliters (μL) of standard/sample were added into each well of a 96-well plate prior to a two-hour (h) incubation. After removing the liquid, 100 μL of horseradish peroxidase avidin (HRP-avidin) was added to each well, and incubation continued for one hour. After washing, 90 μL of 3,3′,5,5′-tetramethylbenzidine (TMB) substrate was added into each well and incubated for another 15–30 min. The plate was next read at 450 nanometers (nm) using a M200 PRO multimode microplate reader (Tecan Group Ltd., Männedorf, Switzerland) within 5 min of adding 50 μL of stop solution into each well. For absolute YKL-40 quantifications, data sets were exported to Excel 2021 (Microsoft Corporation, Washington, DC, USA), triplicates were averaged, and the standard dilutions were fit to a linear curve.

### 2.3. Statistical Analysis

The homogeneity of subjects with severe COPD and very severe COPD in terms of age and sex was assessed using a Mann–Whitney U test and a Chi-square (χ^2^) test, respectively [25]. Inter-group differences in smoking status (ever smokers vs. never smokers) were determined using a χ^2^ test, with current smokers and ex-smokers being pooled together as ever smokers. Frequencies of different classes of ever smokers among the two COPD strata were compared using a similar approach. Next, a *t*-test was applied to assess inter-group differences in smoking duration (as pack-years) for ever smokers.

The neutrophil-to-lymphocyte ratio (NLR) was calculated as the simple ratio between the neutrophil counts and lymphocyte counts. The measured values for serum YKL-40 levels, ALLC, ANC, neutrophil percentage, ALC, lymphocyte percentage, NLR, AEC, eosinophil percentage, AMC, monocyte percentage, ABC, basophil percentage, hemoglobin levels, and hematocrit concentrations were compared between individuals with severe COPD and those with very severe COPD using Mann–Whitney U tests. The study population was then partitioned into two groups based on the median serum YKL-40 value of the overall population. The stratification of study population based on a threshold defined a posteriori is frequently used in pilot trials dealing with novel biomarkers for which there is no well-defined reference range [25]—as in the case of YKL-40 [8,11,12,13,14]. Division of patients into two strata is also commonly employed in exploratory pilot investigations due to a small to moderate sample size (20–50 participants) because it enables a reliable sample size calculation for future large studies [26]. Moreover, combining severe and very severe COPD patients can yield a more homogenous study population, reducing variability in disease severity. This can make it easier to detect meaningful effects if an intervention is expected to have a similar impact across these stages [25,26]. All statistical analyses were conducted using the Statistica version 8 software (StatSoft Inc., Tulsa, OK, USA). Statistical significance was defined at *p* less than 0.05.

## 3. Results

### 3.1. Serum Parameters by COPD Stage (Severity)

After sex- and age-matching, 23 subjects with severe COPD and 24 subjects with very severe COPD were recruited from the initial pool of patients. No mortalities were recorded during the trial. Sociodemographic information (age, gender, and smoking history) according to the COPD stage are shown in Table 1. No significant differences in median age were observed between these categories of subjects (Mann–Whitney U test, *p* = 0.975). Data for gender distribution were also similar between these strata (χ^2^ test, *p* = 0.679), with twice as many male patients than female patients (Table 1).

Smoking had a similar incidence in adults with severe COPD and those with very severe COPD (χ^2^ test, *p* = 0.964), with 10 times as many ever smokers (both current smokers and ex-smokers) than never smokers (Table 1). The frequency of ex-smokers was higher in the latter strata (12 ex-smokers and 10 current smokers for very severe COPD and 8 ex-smokers and 13 current smokers for severe COPD) but did not reach statistical significance (χ^2^ test, *p*  =  0.279). There were also no significant differences (*t* test, *p* = 0.202) in the duration of smoking between ever smokers with very severe COPD (22.5 ± 8.5 pack-years) and those with severe COPD (19.5 ± 6.5 pack-years).

Median values (with lower and upper quartiles) for YKL-40, ALLC, ANC, neutrophil percentage, ALC, lymphocyte percentage, NLR, AEC, eosinophil percentage, AMC, monocyte percentage, ABC, basophil percentage, hemoglobin levels, and hematocrit in patients with severe COPD and patients with very severe COPD are summarized in Table 2. The reference range for hematological parameters are given in the same table.

CBC analysis revealed subtle perturbations in the blood profile of patients with advanced COPD. Thus, ALLC was well above the normal values in healthy patients. In contrast, the measured values for ALC and lymphocyte percentage were below the normal range. However, no significant inter-group differences were found for the blood parameters analyzed (Table 2; Mann–Whitney U tests, *p* ≥ 0.131).

### 3.2. Variables Associated with High YKL-40 Levels

**Figure 1:**
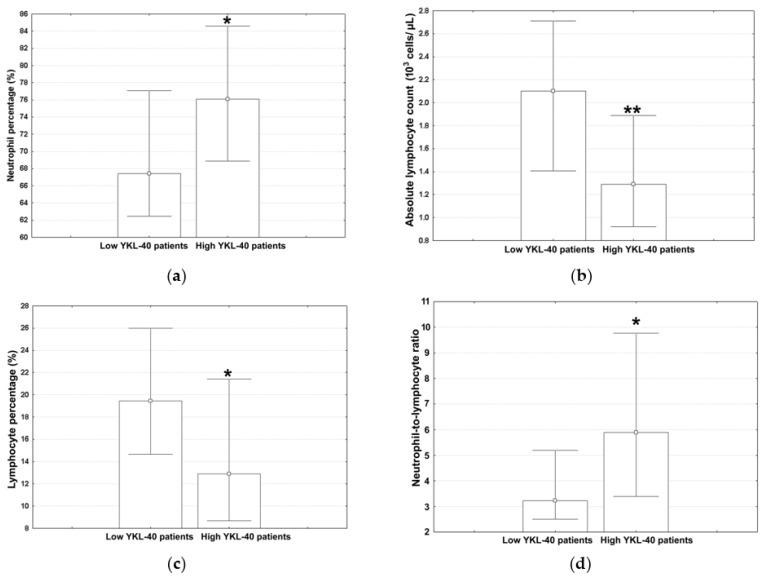
The measured values for (**a**) neutrophil percentage, (**b**) ALC, (**c**) lymphocyte percentage, and (**d**) NLR in different YKL-40 strata. Marked values (*) indicate significant differences as compared to low YKL-40 patients (Mann–Whitney U tests, **—*p* < 0.01, *—*p* < 0.05).

**Figure 2:**
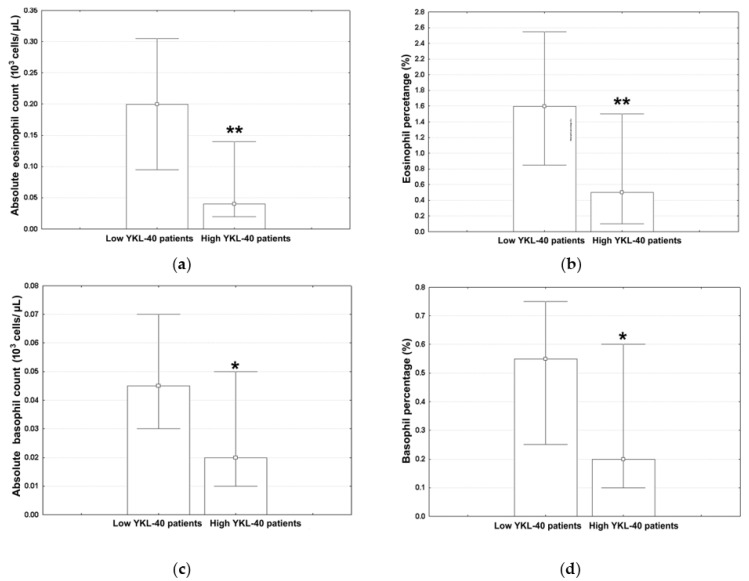
The measured values for (**a**) AEC, (**b**) eosinophil percentage; (**c**) absolute basophil count, and (**d**) basophil percentage in different YKL-40 strata. Marked values (*) indicate significant differences as compared to low YKL-40 patients (Mann–Whitney U tests, **—*p* < 0.01, *—*p* < 0.05).

The subjects were split into two groups using the median of serum YKL-40 of the overall population (3934.5 pg/mL) as a cut-off point. The median values (with lower and upper quartiles) for the ALLC, ANC, ALC, AMC, monocyte percentage, hemoglobin levels, and hematocrit in low YKL-40 patients and high YKL-40 YKL patients are shown in Table 3. The corresponding values for the neutrophil percentage, ALC, lymphocyte percentage, and NLR are shown in Figure 1a–d; those for the AEC, eosinophil percentage, ABC, and basophil percentage are shown in Figure 2a–d.

The neutrophil percentage in subjects from the highest YKL-40 quartile showed a significant increase relative to the low YKL-40 adults (Figure 1a; Mann–Whitney U test, *p* = 0.024). This is in contrast with the results obtained for ALC (Figure 1b; Mann–Whitney U test, *p* = 0.007) and the lymphocyte percentage (Figure 1c; Mann–Whitney U test, *p* = 0.025). The measured values for NLR (Figure 1d; Mann–Whitney U test, *p* = 0.028) showed a trend similar to that seen for the neutrophil percentage.

The AEC (Figure 2a; Mann–Whitney U test, *p* = 0.001) was significantly reduced in high YKL-40 subjects. Similar results were obtained for the eosinophil percentage (Figure 2b; Mann–Whitney U test, *p* = 0.002). The same trend was also observed for the basophil-related parameters; that is, the ABC (Figure 2c; Mann–Whitney U test, *p* = 0.021) and the basophil percentage (Figure 2d; Mann–Whitney U test, *p* = 0.014).

## 4. Discussion

The current work provides the first insight into the connection between serum YKL-40 and CBC parameters during AECOPDs in the high-risk cluster of patients with advanced COPD [5]. This expands current knowledge about this research topic, which until now was limited to scarce, disparate data derived from subjects covering all stages of COPD [7,8,15,18]. The results of the present work are hence important for a better understanding of the clinical relevance of serum YKL-40 and CBC parameters as biomarkers for COPD.

The distribution of different smoking classes (never smokers vs. ever smokers) was similar between severe COPD and very severe COPD, as was the number of pack-years of tobacco smoking and the frequency of current smokers and ex-smokers. These data show that the study population was homogeneous not only in terms of age and sex but also with respect to the smoking status, history, and duration. The majority of adults with advanced COPD were ever smokers. This adds to the extensive body of literature supporting the critical role of smoking in the development of COPD [27,28,29]. In the present work, the prevalence of COPD was also higher in Romanian men than in Romanian women. Indeed, epidemiological data compiled from Eurostat (Luxembourg) showed that 30.6% of Romanian men smoked in 2019 vs. only 7.5% of women [30].

The medical literature provides some indication of a link between serum YKL-40 levels and acute exacerbation episodes in patients with pre-existing COPD. Lai et al. (2016) identified a significant correlation between increased YKL-40 expression and AECOPDs, possibly mediated via fibroblast-induced airway remodeling [7]. More recently, Peng et al. (2021) found that elevated YKL-40 is directly associated with the 1-year COPD-related readmission rate [15]. Both these studies included patients with all stages of COPD. The present trial, in contrast, involved only individuals with advanced stages of COPD but focused on serum YKL-40 levels during AECOPDs and not on its prognostic value for exacerbation attacks. The results obtained revealed the absence of significant inter-group differences in the measured values for this glycoprotein. This does not favor the use of serum YKL-40 for separating severe COPD and very severe COPD during AECOPDs.

The observed lack of association between serum YKL-40 and COPD severity may suggest that the link between the aforementioned protein and the pathophysiology of exacerbated COPD is weak. Indeed, COPD is a complex and heterogeneous disease with multiple underlying mechanisms, including chronic inflammation, airway remodeling, and oxidative stress [6,31]. Although YKL-40 is associated with inflammation and tissue remodeling [7,8,11,12,13,14,15], it may not capture the full spectrum of pathophysiological processes at play in COPD exacerbations. Other biomarkers or clinical factors may hence play a more significant role in determining disease outcomes. Since YKL-40 levels correlate directly with COPD severity and exacerbations [7,8,14,15,16], it is, however, plausible that this weak association is related to the fact that only patients with advanced COPD were included in this study. Moreover, serum YKL-40 may serve as an independent biomarker in predicting responsiveness or insensitivity to anti-COPD medications and more exacerbations [8,10].

The most noticeable change in CBC parameters (relative to normal levels) was elevated ALLC (leukocytosis). Lymphocytes were the only type of leukocytes with values outside the reference range; more precisely, below-normal levels (lymphopenia). Both leukocytosis and lymphopenia are often encountered during AECOPD episodes, when the immune response is activated due to an underlying infection or inflammation in the airways [32,33,34,35]. However, these immune responses alone are not specific to AECOPDs and can occur in response to various other infections and inflammatory conditions [6].

Compared to low YKL-40 individuals, high YKL-40 adults had significantly higher neutrophil percentages but similar ANCs during exacerbation episodes. It hence appears that the latter strata displays changes in the percentage of other types of leukocytes rather than quantitative changes related to the neutrophil count. Indeed, data sets for other leukocyte classes support this assumption. Thus, elevated levels of serum YKL-40 were associated with a significant drop in both ALC and lymphocyte percentage. These events can be attributed to several factors. Inflammation-induced recruitment/activation of inflammatory cells during AECOPDs can suppress lymphocyte activity [36,37], causing a decrease in lymphocyte-related blood parameters. It is also plausible that AECOPD-related systemic inflammation and oxidative stress can disrupt the normal functioning of bone marrow or accelerate lymphocyte apoptosis [38,39], leading to a reduction in lymphocyte production.

Enhanced NLR values, as observed here in high YKL-40 patients, reflect a disbalance between neutrophils and lymphocytes, pointing to a pro-inflammatory state [40]. On the other hand, increased serum YKL-40 concentrations reflect ongoing inflammation and tissue damage [8]. As a result, the combination of a high serum NLR and YKL-40 during AECOPDs may indicate a more severe and progressive disease phenotype.

Eosinophil-based parameters measured here in high YKL-40 patients with advanced COPD during acute exacerbations were significantly below those seen for low YKL-40 subjects. The only study connecting serum YKL-40 with eosinophils in the context of AECOPDs has identified, in contrast, a direct association between these parameters. However, it focused on their predictive value for COPD-related readmission rates across all stages of COPD and not on the CBC profile during acute exacerbation episodes of advanced disease [15]. Since relatively higher eosinophils are associated with a reduced risk for AECOPD-related morbidity and mortality [41,42], low YKL-40 subjects might represent a phenotype with less severe disease. This presumption is consistent with clinical data [7,8,11,12,14,15]. With respect to the mechanisms underlying this association, elevated serum YKL-40 may activate inflammatory cells, which in turn will release factors promoting eosinophil recruitment and their retention within the lungs [43], inducing a subsequent decrease in circulating eosinophil levels. It is also plausible that increased levels in the serum of patients with advanced COPD may perturb the eosinophil life cycle, leading to decreased levels in the bloodstream [38]. Furthermore, elevated YKL-40 may promote a pro-inflammatory environment that is less supportive of eosinophil presence [44].

This study also provides pertinent evidence linking basophils to serum YKL-40 in AECOPDs. Such information is currently not available in the medical literature. The significant decrease in both the basophil count and frequency seen in high YKL-40 individuals can be related to several factors. Considering their role in mediating immune responses by migrating to the site of inflammation [45], it is possible that this event reflects the redistribution of basophils from the bloodstream into the lungs [46], leading to a reduction in their circulating levels. In addition, persistent inflammation may yield chronic activation and degranulation of basophils, with the subsequent release of granules leading to a depletion in or the decreased circulation of basophils [47].

Overall, the results of the present study suggest that high serum YKL-40 in patients with advanced COPD and AECOPDs is associated with significantly decreased values for lymphocyte-, eosinophil-, and basophil-related CBC parameters but no changes in neutrophile and monocyte counts. It is generally thought that airway inflammation in COPD is primarily driven by Type 1 immune responses (Th1 response), whereas Type 2 inflammation (Th2 response) is present in definite proportions during stable and exacerbation phases of COPD [48]. From an immunological point of view, the aforementioned pattern of changes in CBC-related parameters is more consistent with a Type 2 inflammation, since it involves a reduction in eosinophils and basophils, which are associated with this type of inflammatory response [48]. In contrast, neutrophils and monocytes, which are more characteristic of Type 1 inflammation, remained unchanged. Therefore, high YKL-40 individuals with advanced COPD are more likely to exhibit Type 2 inflammation rather than Type 1 inflammation in acute exacerbations. The identification of these features illustrate the complexity and heterogeneity of COPD immunopathology, with potential implications for phenotyping (endotyping) and personalized treatment.

Hirano and Matsunaga (2023) recently summarized the current state of knowledge regarding the link between eosinophils and clinical outcomes of COPD [48]. Based on these data, the low YKL-40 group is more likely to display the eosinophilic endotype of COPD, whereas the high YKL-40 group is more likely to display the non-eosinophilic endotype of COPD. The former category of patients tends to exhibit a lower risk profile with a shorter length of hospitalization, reduced morbidity, and a better response to corticosteroid treatment [49]. In contrast, high YKL-40 individuals may benefit more from treatment with antibiotics instead of systemic corticosteroids [50], as low eosinophils are associated with a higher prevalence of bacterial exacerbations [49]. One may hence expect a more severe impairment in the immune response of these patients that is potentially related to immune cell exhaustion and leads to a more severe risk profile and clinical outcomes. However, it is important to note that inflammation in COPD is complex and multifactorial, and the specific cytokine profile and immune response may vary among individuals [6,10].

The findings of this trial are subject to several limitations. The exploratory nature of this pilot study and the moderate sample make it difficult to generalize these findings. The COVID-19 pandemic also did not allow us to obtain blood samples during the stable phase of COPD, hence the lack of control sampling during the stable phase of the same patients. Longitudinal studies with a larger sample size and measurements conducted during both stable and exacerbation periods are therefore necessary to validate these results. However, this type of study is helpful in detecting meaningful associations between different variables, thus guiding the formulation of hypotheses regarding the relationships observed [51]. Another drawback is the fact that this investigation did not assess the number and frequency of different types of leukocytes in the sputum or lungs of COPD patients during exacerbation episodes. These individuals often exhibit altered leukocyte profiles in these body matrices [52], and as a consequence, such measurements may help confirm and explain the differences noticed here in different YKL-40 strata. The diagnosis of COPD relied on spirometric measurements, but we were not able to show these values given the lack of electronic database of spirometry. Nonetheless, this study was conducted in a specialized department of the hospital, with COPD being diagnosed by a pulmonary specialist (in collaboration with other academic pulmonary specialists) and hence with very little suspicion of misdiagnosis. Moreover, these findings may not be applicable to other populations and races, as there is relevant indication for racial differences in COPD phenotype and incidence [53].

Notwithstanding these limitations, this study offers some intriguing insights into the connection between YKL-40 and CBC parameters in advanced COPD during acute exacerbation episodes. A key strength is that it conducted a comprehensive examination of multiple types of leukocytes rather than single levels. Another strength of the present study is the fact that the subjects were age- and sex-matched before the start of the study. This allowed us to avoid the risk of having to run the analysis with too many strata but a reduced sample size [51]. In contrast, most studies on YKL-40 and COPD used statistical age adjustment to limit the bias of age for the cohorts compared (e.g., [11,12]). This a posteriori adjustment approach has several drawbacks with respect to pilot studies. One is overfitting, which occurs when the model fits the noise or random variations in the data rather than capturing the underlying true patterns. Overfit models are likely to perform poorly when applied to new, unseen data, like those derived from exploratory studies [51]. Another disadvantage is selection bias [51]. Statistical adjustment techniques often assume that all relevant variables are included in the adjustment model. However, if important variables are omitted or not properly accounted for, the adjustment may introduce selection bias. Selection bias occurs when the adjustment fails to address systematic differences between the adjusted groups, leading to biased estimates [51]. Since all biochemical analyses were performed for patients during the exacerbation periods, it is also plausible that their CBC profiles and YKL-40 levels were more deranged vs. stable periods [35,39]. As a result, the magnitude of changes in these parameters may be higher and hence more relevant to understanding the immunopathological connection between YKL-40 and CBC in COPD.

To sum up, the following main clinical implications were derived from these results of the present investigation. First, the multidimensional shift observed here in the leukocyte profile during AECOPDs could have clinical implications for tailoring treatment strategies. For example, patients with high serum YKL-40 concentrations may benefit from antibiotic interventions, while those with low YKL-40 levels may need corticosteroid therapy. Second, measuring YKL-40 levels in advanced COPD individuals during exacerbations might provide new insights into the severity and prognosis of AECOPDs. That is, high YKL-40 subjects may display a higher risk of exacerbation-related complications, thereby requiring more intensive monitoring and treatment. Third, interventions targeting YKL-40 or pathways in which it is involved might be beneficial in managing AECOPDs, allowing the development of drugs or therapies that modulate YKL-40 expression or function.

Investigating the underlying mechanisms by which YKL-40 influences leukocyte profiles is an important future research direction. This should involve laboratory-based experiments to decipher the molecular pathways involved and how they relate to the immune response in COPD exacerbations. In addition, clinical studies can be designed to identify the effectiveness of therapies for YKL-40 or related pathways in AECOPDs. These trials should determine if modulating serum YKL-40 concentrations leads to improved outcomes, reduced exacerbation frequency, or enhanced lung function. Research could also focus on refining the criteria for stratifying COPD patients based on YKL-40 levels. Identifying specific YKL-40 thresholds/patterns of response to treatment may help guide personalized management strategies. Furthermore, understanding the relationship between YKL-40 levels and comorbidities commonly associated with COPD, such as cardiovascular disease or osteoporosis, could provide insights into the broader systemic effects of this protein.

## 5. Conclusions

In conclusion, patients with advanced COPD but different serum YKL-40 levels displayed different leukocyte profiles during acute exacerbations. More precisely, high YKL-40 subjects showed a significantly increased neutrophil percentage and NLR but significantly lower lymphocyte-, eosinophil-, and basophil-related parameters compared to the low YKL-40 individuals. These multidimensional, YKL-40-associated changes in the leukocyte profiles of patients with advanced COPD may be useful for personalized treatment and a better understanding of AECOPD immunopathology.

## Figures and Tables

**Table 1:** Sociodemographic characteristics and smoking history according to COPD severity.

COPD Stage	Age	Sex		Smoking Status	
		Male	Female	Ever Smoker	Never Smoker
Severe COPD	66 (61; 72)	15 (65.21%)	8 (34.79%)	21 (91.30%)	2 (8.70%)
Very severe COPD	66.5 (57; 70)	17 (70.84%)	7 (29.16%)	22 (91.67%)	2 (8.33%)

Data for age are shown as median values with lower and upper quartiles (in parentheses). Data for sex and smoking status are given as absolute values and the corresponding percentages (in parentheses).

**Table 2:** Measured values for selected blood parameters in the study population.

Characteristic	Severe COPD (*n* = 23)	Very Severe COPD (*n* = 24)	Reference Range
YKL–40	3960.5 (3027.5; 4947.25)	3925.5 (2924.25; 4904.5)	
ALLC (10^3^ cells/μL)	11.36 (9.34; 14.31)	9.35 (7.38; 11.77)	1–4
ANC (10^3^ cells/μL)	8.49 (6.71; 10.49)	7.13 (4.93; 8.41)	2–8
Neutrophil percentage (%)	71 (62.7; 81)	70.1 (65.4; 79.5)	45–80
ALC (10^3^ cells/μL)	1.75 (1.05; 2.91)	1.54 (0.94; 2.04)	4–10
Lymphocyte percentage (%)	17 (10.6; 20.9)	17.3 (11.05; 23.55)	20–55
NLR	3.99 (2.56; 7.94)	3.90 (2.90; 7.35)	
AEC (10^3^ cells/μL)	0.12 (0.02; 0.27)	0.10 (0.03; 0.19)	0.05–0.7
Eosinophil percentage (%)	1.4 (0.2; 2.1)	1.0 (0.35; 1.65)	0–7
AMC (10^3^ cells/μL)	1.05 (0.71; 1.30)	0.85 (0.58; 0.97)	0.3–1
Monocyte percentage (%)	7.70 (6.50; 10.70)	8.25 (6.55; 10.35)	0–15
ABC (10^3^ cells/μL)	0.04 (0.01; 0.08)	0.04 (0.02; 0.06)	0–0.2
Basophil percentage (%)	0.4 (0.1; 0.7)	0.4 (0.2; 0.6)	0–2
Hemoglobin (g/dL)	13.5 (12; 14.2)	14.10 (12.4; 15.55)	12.6–17.4
Hematocrit (%)	41.6 (38.5; 43.6)	43.95 (37.8; 49)	37–51

Data are given as median values with lower and upper quartiles (in parentheses). ALLC, absolute leukocyte count; ANC, absolute neutrophil count; ALC, absolute lymphocyte count; NLR, neutrophil-to-lymphocyte ratio; AEC, absolute eosinophil count; AMC, absolute monocyte count; ABC, absolute basophil count.

**Table 3:** Measured values for selected blood parameters in low and high YKL-40 patients.

Characteristic	Low YKL-40 Patients (*n* = 24)	High YKL-40 Patients (*n* = 23)
ALLC (10^3^ cells/μL)	10.81 (8.05; 13.04)	10.57 (8.16; 13.71)
ANC (10^3^ cells/μL)	7.25 (5.25; 8.70)	7.91 (5.79; 10.81)
AMC (10^3^ cells/μL)	0.87 (0.70; 1.08)	0.80 (0.63; 1.04)
Monocyte percentage (%)	8.10 (6.70; 9.65)	7.70 (6.10; 12.70)
Hemoglobin (g/dL)	14.05 (12.75; 15.25)	13.50 (12.10; 14.20)
Hematocrit (%)	43.40 (38.00; 42.75)	42.20 (38.30; 44.50)

Data are given as median values with lower and upper quartiles (in parentheses). ALLC, absolute leukocyte count; ANC, absolute neutrophil count; AMC, absolute monocyte count.

## Data Availability

All the data generated or analyzed during this study are included in this published article.
